# Nanoscale cryptography: opportunities and challenges

**DOI:** 10.1186/s40580-015-0052-8

**Published:** 2015-11-02

**Authors:** Massoud Masoumi, Weidong Shi, Lei Xu

**Affiliations:** Department of Computer Science, University of Houston, 501 Philip G. Hoffman Hall, Houston, TX 77204-3010 USA

**Keywords:** Nanoelectronics, Cryptography, hardware implementation, Side-channel attacks

## Abstract

While most of the electronics industry is dependent on the ever-decreasing size of lithographic transistors, this scaling cannot continue indefinitely. To improve the performance of the integrated circuits, new emerging and paradigms are needed. In recent years, nanoelectronics has become one of the most important and exciting forefront in science and engineering. It shows a great promise for providing us in the near future with many breakthroughs that change the direction of technological advances in a wide range of applications. In this paper, we discuss the contribution that nanotechnology may offer to the evolution of cryptographic hardware and embedded systems and demonstrate how nanoscale devices can be used for constructing security primitives. Using a custom set of design automation tools, it is demonstrated that relative to a conventional 45-nm CMOS system, performance gains can be obtained up to two orders of magnitude reduction in area and up to 50 % improvement in speed.

## Background

Since the beginning of the seventies, microelectronics industry has followed Moore’s law, doubling processing power every 18 months. This performance increase has been obtained mainly by decreasing the size of circuit features obtained by optimization and improvement of existing technology. Based on the SIA (Semiconductor Industry Association) roadmap, it seems likely that CMOS will remain the mainstream of IC technology even after 2014 and has many years to go (http://www.semiconductors.org/, http://www.itrs.net/). However, it is clear that the Moore’s Law exponential increases in density and performance cannot be maintained for ever and with ongoing shrinking dimensions, a MOS transistor will ultimately cease to operate as a proper field-effect-transistor. The main reason is that at gate length around or below 10 nm, the sensitivity of transistor parameters, (most importantly, the gate voltage threshold) of silicon field-effect transistors (MOSFETs) to inevitable fabrication spreads grows exponentially. As a result, the gate length should be controlled with a few-angstrom accuracy, far beyond even the long-term projections of the semiconductor industry. On the other hand, the technical limit to interconnect complexity is much harder to define [1]. There is a great preference in the semiconductor industry for the system-on-a-chip with as many different functional silicon-based (and perhaps other) devices on one silicon chip. Nanotechnologies which may be integrated onto a CMOS chip would be the preferred route [2, 3]. If the nanoelectronic field wants to mature to this stage, there is a necessity to bring novel devices more on a par with CMOS by developing the necessary fabrication processes, simulation tools and design rules that are required for any industrial electronic manufacturing process. As a result, researchers in any one particular area need to reach beyond their expertise in order to appreciate the broader implications of nanotechnology, and learn how to contribute to this exciting new field. One of the most important and valuable potential application areas may be information security and integrated implementation of cryptographic systems [4–10]. Indeed, the main threat to a cryptographic token in the real world is not the cryptanalysis of the actual algorithm, but rather the exploration of weaknesses of the implementation of the algorithm in the real world [11]. These are mainly due to the inherent weaknesses of CMOS technology in which CMOS transistors leak information related to sensitive information when they are switched “ON” or “OFF” [12]. However, nanoelectronic may change this situation. The new hybrid technology paradigm will certainly require rethinking of the current circuit architectures. This work describes a radically different yet efficient approach based on the combination of CMOS circuits along with nanoelements for the implementation of the security circuits which may provide a significantly improved performance [13]. Instead of insisting on existing solutions, we focus on some new solutions that may have the highest potential for conceptual breakthroughs. Based on this fact, we are proposing that digital hybrid CMOS-Nanoelectronic reconfigurable architecture [14] is a potential optimum platform to realize encryption algorithms. It will be demonstrated that such a design result in a circuit which is faster and strikingly denser than its CMOS counterpart. To better demonstrate the capabilities of the proposed approach, we have implemented the basic modules of the Secure Hash Algorithm [15] on cell-FPGA-like hybrid semiconductor-nanowire-nanodevice platform which combines a CMOS transistor stack and two levels of parallel nanowires using some already available CAD-tools. We believe that this work will lead to a paradigm shift incorporating security fully into the design and development of future generations of nanoscale computing hardware. The contribution of this paper is as follows:

1. This paper presents the first crossbar based nanoscale computing platform (nano-architecture) for the implementation of encryption algorithms. This uniform array of nanowires in a multi-layer CMOS-Nano crossbar structure provides manufacturability by regularity, reliability (fault tolerance) by reconfigurability, and performance by logic density. Although, some works have addressed the use of nanowire crossbar architecture for logic implementation [2] but their performance cannot be easily evaluated and compared to MOSFET FPGAs.
2. So far, mainly 128-bit key encryption has been designed and implemented by CMOS technology, primarily due to area, speed and power consumption problems associated with the implementation of the encryption algorithm with longer keys. The results we have obtained demonstrate that longer keys can be easily realized by hybrid CMOS-Nano FPGA architecture, making the implementation much more robust against unauthorized deciphering and cryptanalytic attacks.

The reminder of paper is organized as follows: Section II briefly explains reconfigurable hybrid CMOS/Nano technology. Section III illustrates the Secure Hash Algorithm very briefly. Section IV presents the performance results of the implementation of secure hash algorithm basic modules on hybrid CMOS/Nano platform. Finally, in the conclusion we briefly summarize the results of our discussions.

### Reconfigurable hybrid CMOS/nanodevice circuits

**Fig. 1:**
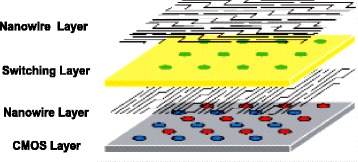
General schematics of a mixed CMOS-Nano system

**Fig. 2:**
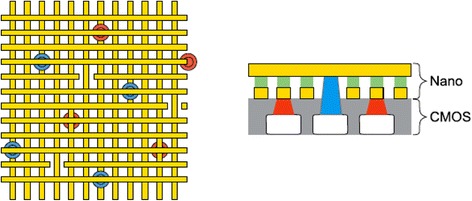
Left: crossing nanowires separated by a configurable nanojunction layer form ‘junctions’ that may be electrically configured as electronic devices. Right: nanowire crossbars connected to a CMOS chip via metallic ‘pins’ on the CMOS surface [28]

Traditional existing microelectronic-based approaches might not able to meet all the performance requirements because of the long term costs and the inherent limitations of CMOS technology [16]. So far, mainly the meaning of nanoscale circuits has been the same CMOS circuits that have been smaller. However, to improve the performance of integrated circuits other emerging and paradigms are needed. A feasible yet efficient scenario is the integration of silicon with nanoelectronics, i.e., a mixed CMOS/nano system. This approach would allow a smooth transition and permits leveraging the beneficial aspects of both technologies. Currently, it is not possible to make an electronic circuit by using only nanoscale devices, but rather combining it with CMOS circuits may be a better idea [17]. The possibility of mixed CMOS/Nano circuits permits using the best aspects of both technologies simultaneously, while the undesired aspects of a technology can be compensated by the partner technology. Hence, Instead of completely replacing the CMOS technology, the common belief is that the future chips for nanotechnology should be built as a hybrid using both CMOS and nanomaterials (such as CarbonNanoTube bundle interconnects and nanotube/nanowire crossbar memories), thus taking advantages of both mature CMOS technology and novel advances in nanotechnology. The basic idea for such circuits is to combine the advantages of current CMOS technology including flexibility and reasonable fabrication yield with nanoscale devices, assembled on a pre-fabricated nanowire fabric, enabling very high function density at modest fabrication cost. Such architectures allow for significant design versatility. For example, while nano portion is restricted to regular structures, the CMOS portion can be used for the implementation of any arbitrary logic circuit. Perhaps one of the most promising structure for such circuits is an FPGA-like architecture combining a CMOS stack and two-level nanowire crossbar with nanodevices formed at each nanowire cross point together with the ability to reconfigure the circuits around nanodevices defects. Such reconfiguration is essential for any mixed CMOS-Nano system because the lack of enough alignment accuracy and also due to the fact that such a fabrication can hardly provide 100 % yield. It has been shown that such architecture is defect tolerant and even with a high degree of defect rate can provide much better performance in terms of area and speed at acceptable power consumption when compared to circuits which use CMOS alone [18, 19]. These circuits work with two-terminal nanodevices whose are electrically activated or deactivated at the cross-points of the mesh and their fabrication is substantially less challenging than their three-terminal counterparts. Of course, the limited functionality of two terminal devices is compensated by transistors of the CMOS subsystem. This is accomplished by CMOS cells which are accessible through column and row lines. These nanodevices are generally resistive junctions with hysteretic switching behaviors. They are reprogrammable and can be reconfigured to be either turned-off or turned-on. Depending on the materials, some devices will also have hysteretic diode-like switching behavior similar to one resistor in series with one diode. The original structure considers junction nanodevices with diode behaviors, which can be used as a memory element or part of the logic gate for field-programmable gate array applications. The general schematic for this architecture is shown Fig. 1. Fig. 2 represents an improved version of this figure in which each crossbar ‘junction’ is generally hypothesized to be an electrically configurable or reconfigurable device. The simplest being may an anti-fuse that is currently being widely used in the state-of-the-art chip manufacturing industries [20]. A positive voltage drop across an antifuse junction might drive it into a low-impedance state, while a negative voltage drop might return it to a high-impedance state. Metallic pins on the surface of the chip connect down into CMOS gates and provide contact points for electrically attaching nanowires in the crossbar. It is necessary to mention that the ‘crossbar’ nanowire structure does not need 100 % alignment with respect to the CMOS subsection and a shift of the nanowire/nanodevice subsystem by one nanowiring pitch with respect to the CMOS base does not affect the circuit properties. This problem is solved using area-distributed interfaces. The idea of achieving CMOS-to-nano interface without any ‘overlay’ alignment using precisely angled cuts, is suggested in [21].

**Fig. 3:**
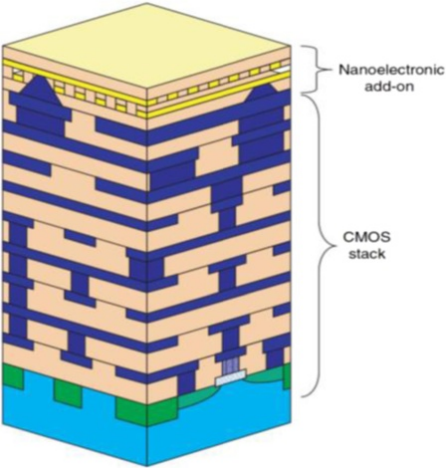
General schematics of a hybrid CMOS/Nano circuit with area distributed interfaces [29]

**Fig. 4:**
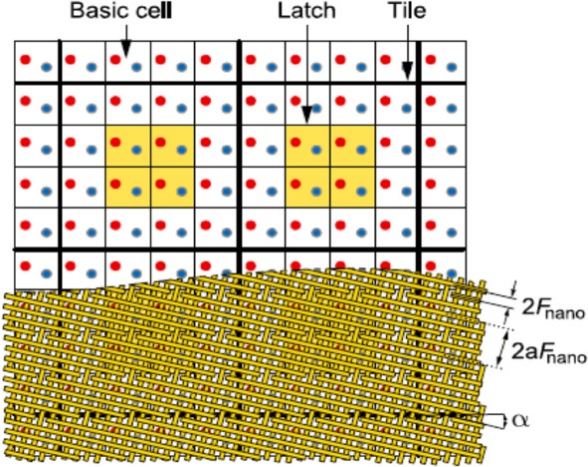
Schematic of three-cell Hybrid CMOS-Nano Fabric [29]

Figure 3 shows the general schematics of a hybrid CMOS/Nano circuit with area distributed interfaces. A hybrid CMOS-Nano basic cell consists of an inverter and two pass transistors connected to two pins (with different heights) serving as the cell input and output. Each square houses a CMOS inverter (or a NAND gate) connected to one input pin (for reading a signal driven from a nanowire) and one output pin (for driving a signal from a gate to a nanowire). The input and output of each gate are connected to the nanowires via connection pads shown by blue and red dots in Fig. 4. The bottom wire mesh which makes connections to the inputs is shown by green while the top wires are shown by yellow and provide connections to the outputs. There is a nanodevice placed at each crosspoint between the bottom and top nanowire meshes. The interesting proposed alignment of nanowires with respect to underlying CMOS cells, which is rotated by a certain angle, makes it possible to address each and every nanodevice. The CMOS row signals are used to program the nanodevices through pass transistors that are controlled by the columns signals. In other words, we are able to access each element and turn them “ON” and create a connection between the top and bottom wire at that point or we can choose to leave it “OFF”, which then acts as an open circuit. It is easy to observe that each and every nanodevice can be addressed by proper choice of two CMOS cells and by using this technique, we are able to implement combinational and sequential logic gates such as NAND, NOT, XOR, Flip-Flops.

**Fig. 5:**
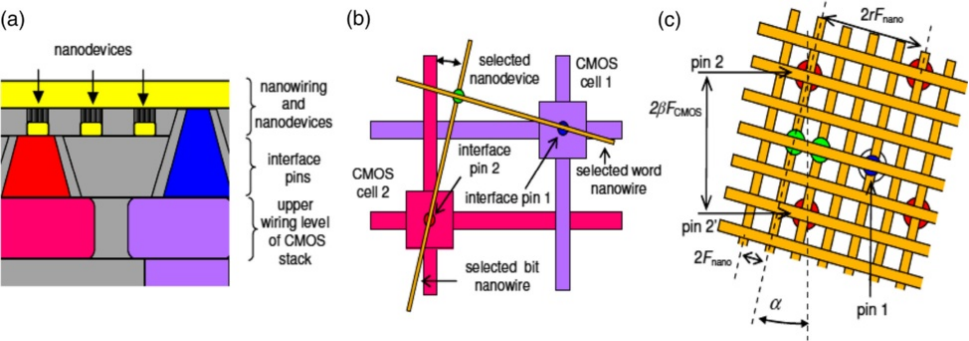
Low level structure of hybrid CMOS/Nano system (**a**) schematic side view; (**b**) the idea of addressing a particular nanodevice, and (**c**) zoom-in on several adjacent interface pins to show that any nanodevice may be addressed via the appropriate pin pair (e.g., pins 1 and 2 for the leftmost of the two shown devices, and pins 1 and for the rightmost device) [29]

**Fig. 6:**
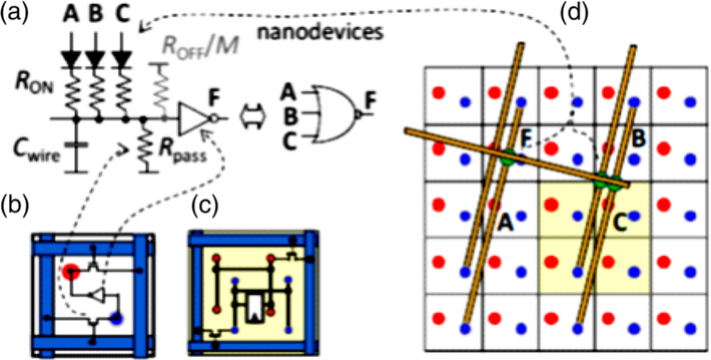
Hybrid CMOS-Nano FPGA circuits (**a**) The idea of diode NOR logic; (**b**) basic inverter and (**c**) latch CMOS cells; for clarity, panel (**d**) shows only nanodevices and nanowires participating in the NOR gate demonstrated on panel (**a**) [29]

**Fig. 7:**
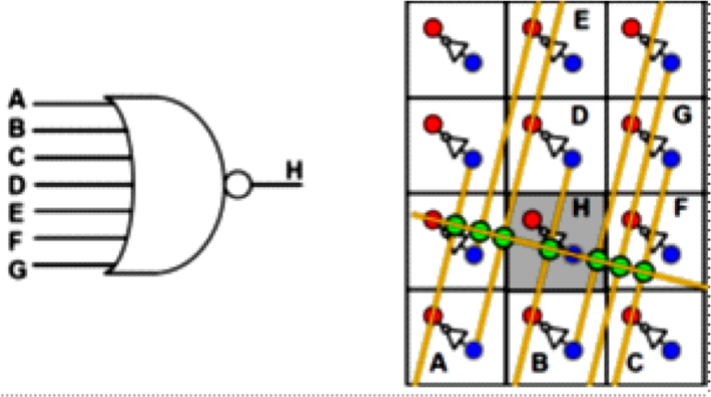
Logic and routing primitives of hybrid CMOS-Nano FPGA, example of the implementation of a 7-input NOR gate. Only active nanodevices are shown [29]

One of the key feature of crossbar hybrid CMOS-Nano circuits is a tilt of the nanowire nanoscale crossbar relative to the CMOS circuitry, which allows to match the pitch 2*F*_*CMOS*_ of the CMOS system and that 2*F*_*nano*_ of the nanowire crossbar. Figure 5 clarifies the subject. Figure 5(a) shows the schematics of hybrid CMOS-Nano system. As it is seen in Fig. 5(c), the interface pins of each type (reaching to either the lower or the upper nanowire level) are arranged into a square array with side 2*βF*_*CMOS*_, where *β* is a dimensionless factor of the order of one that depends on the CMOS cell complexity. The nanowire crossbar is turned by angle α = arcsin (*F*_*CMOS*_/*βF*_*nano*_) relative to the CMOS pin array. Hence, by activating two pairs of perpendicular CMOS wires, we can select two individual nanowires and program a single nanodevice at their crosspoint (Fig. 5(b)) to connect or disconnect these two nanowires This not only makes each nanodevice individually accessible from CMOS subsection (even if *F*_*nano*_ ≪ *F*_*CMOS*_) but also makes the system robust against small shifts of nanowire section. Indeed, studies of [22] shows that at the optimal choice of the pin tip diameter (equal to *F*_*nano*_), there is only one specific mutual position of the pins and crossbar (in each of two perpendicular directions), at which the connection between these two subsystems is imperfect, while even a small shift from that position restores the proper connectivity. This structure also allows us to use the high drive strength CMOS transistors to buffer and restore each nanowire output signal. The hybrid CMOS-Nano wired logic depends on the voltage divider between the junction switch (modeled as a resistor *R*_*on*_) and the pass transistor (modeled as a resistor *R*_*PASS*_) in order to provide a suitable voltage level to the input of the inverter. Figure 6 shows the NAND/buffers/flip-flop cells in the hybrid CMOS-Nano architecture. For clarity, Fig. 7 shows the implementation of a 7-input NOR gate from another perspective in which active nanodevices are shown in green while unused nanodevices are not shown. Please notice that this multi inputs NOR gate is implemented by only one minimum size inverter and several nanoelements while for the implementation of the same function in CMOS technology several NMOS and PMOS transistors are needed. This is the main reason that hybrid CMOS-Nano circuits are far smaller than their CMOS counterparts.

It should be mentioned that the enormous density of two-terminal nanodevices can hardly be used without reliable individual contacts to each of them. This is why the fabrication of wires with nanometer-scale cross-section is another fundamental problem of nanoelectronics. The currently available photolithography and patterning methods, and even their rationally envisioned extensions, will hardly be able to provide a few nanometer resolution. In addition, the scaling of the pitch (*F*_*nano*_) below 3 nm value would not be practical because of the quantum mechanical tunneling between nanowires. Hence, scaling down of these circuits in nano section will be limited by some fundamental problems. However, as will be demonstrated, hybrid CMOS-Nano circuits provide higher degree of integratability compared to their CMOS counterpart with the same feature size and design rules.

### SHA-512 Logic

**Fig. 8:**
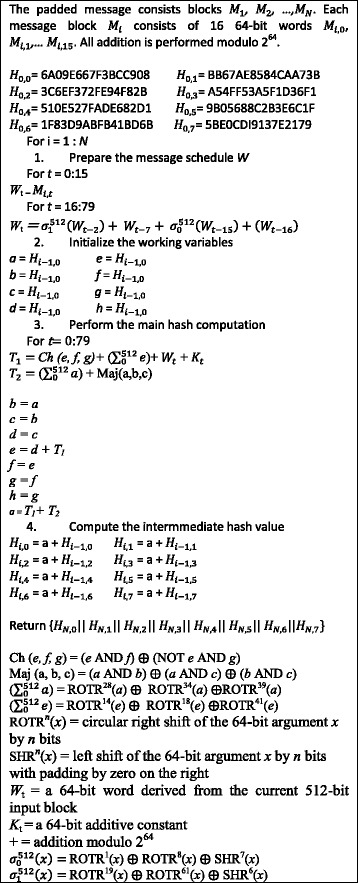
SHA-512 Logic [23]

In order to better demonstrate the effectiveness of the proposed approach and to compare the performance of hybrid CMOS-nanowire-nanoelement crossbar with conventional CMOS circuits, we have implemented the building modules of secure hash algorithm using both regular CMOS and hybrid CMOS-Nano circuits. In this section we briefly describe the secure hash algorithm. The algorithm takes as input a message with a maximum length of 2^128^ and produces as output a 512-bit message digest. The input is processed in 1024-bit blocks. Each round takes as input the 512-bit buffer value, abcdefgh, and updates the contents of the buffer. At input to the first round; the buffer has the value of the intermediate hash value, *H*_*i* ‐ 1_. Each round *t* makes use of a 64-bit value *W*_*t*_, derived from the current 1024 bit block being processed (*M*_*i*_). Each round also makes use of an additive constant *K*_*t*_, where 0 ≤ *t* ≤ 79 indicates one of the 80 rounds. The SHA-512 algorithm has the property that every bit of the hash code is a function of every bit of the input. The complex repetition of the basic function produces results that are well mixed; that is, it is unlikely that two messages chosen at random, even if they exhibit similar regularities, will have the same hash code. Unless there is some hidden weakness in SHA-512, which has not so far been published, the difficulty of coming up with two messages having the same message digest is on the order of operations, while the difficulty of finding a message with a given digest is on the order of operations. The algorithm has four basic modules: *Round Function, Round Operation, Final Round Addition and Round Word Computation.* Figure 8 represents the overall processing of a message to produce a message digest [23].

## Methods

Using a completely custom design automation flow, we first mapped the circuits in .BLIF format on the two-cell hybrid CMOS-Nano FPGA fabric tile array without defect and then with 10 % and 30 % defect rate (https://www.ece.ucsb.edu/~strukov/papers/2007/CMOLCAD2007.pdf, http://www.eecg.utoronto.ca/vpr/) [28, 29]. To study how those defects impact the yield and critical path timing, we compiled each of the four mentioned building blocks, varying the ‘stuck at open’ nanojunction defect probability from 0–0.3. In order to have a fair comparison with CMOS FPGA, the results for different circuits are obtained for the CMOS-Nano FPGA fabric with exactly the same operating conditions and physical structure for all the circuits, thus enabling a fair comparison with CMOS FPGA. For this comparison, the same benchmark circuits have been synthesized into cluster-based island-type logic block architecture. This was done with T-VPack and VPR tools using the architecture designed for the optimal area-delay product, specifically the cluster size of 4, 4-input LUTs, and the VPR’s default architecture file (4x4 LUT-sanitized arch.) with technology parameters corresponding to the 45 nm CMOS process (http://www.eecg.utoronto.ca/vpr/). We first found the worst case segment width required to route every circuit successfully. Then, using architecture with such segment width we mapped and routed all circuits, and then extracted their delay and area (for the optimistic case of buffer sharing). The full delay of the considered circuits was calculated from the critical path, which was found after circuit placement and global routing. The delay of 1-input NOT gate which is the basic logic primitive of hybrid CMOS-Nanoelement circuit turns out to be about 40 ps based on Eq. (1).

1 $$ \tau = \ln \left(2\mathrm{I}\right)\times \left({c}_{wire}\ {R}_{ON}/\mathrm{D}\right)\times \left({V}_{in}/{V}_{DD}\right) $$

## Results and Discussions

**Fig. 9:**
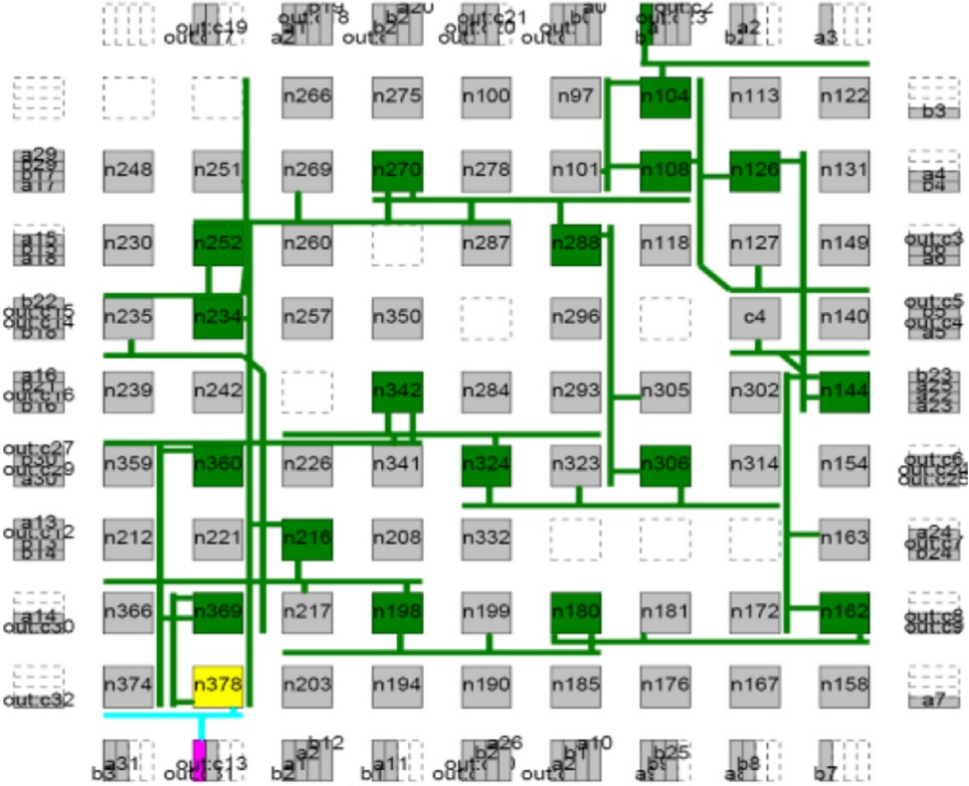
Critical path of the implementation of Final Round Addition on (9 + 2) × (9 + 2) CMOS FPGA block is represented in green

**Fig. 10:**
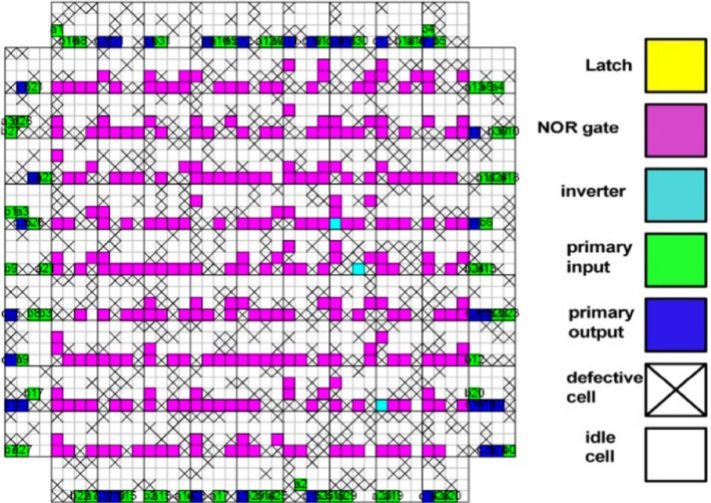
Placement of Final Round Addition in .BLIF format after reconfiguration with present of 30 % defective cells mapped on a 9 × 9 hybrid CMOS-Nano FPGA fabric

**Fig. 11:**
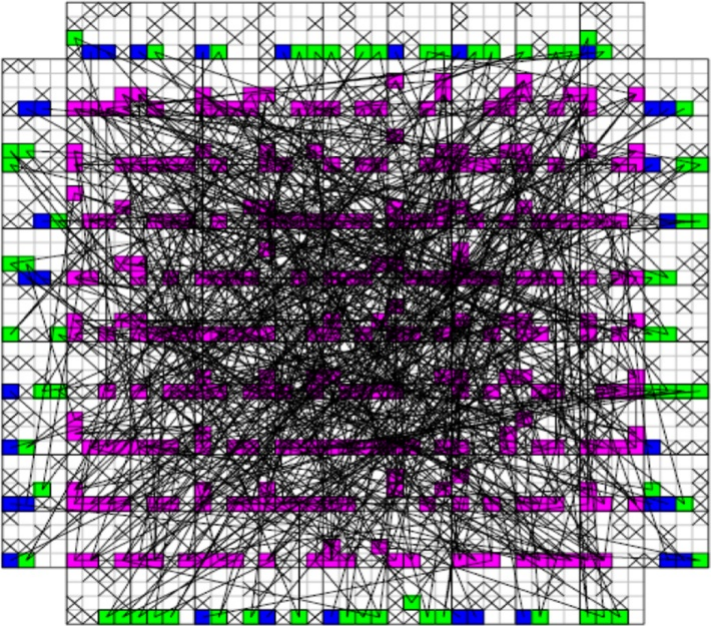
Initial routing of Final Round Addition in .BLIF format with presence of 30 % defective cells

**Fig. 12:**
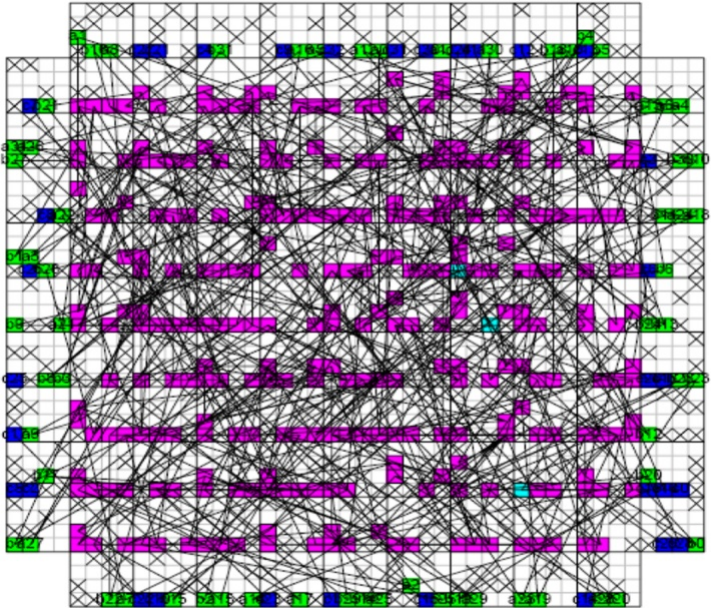
Final routing of Final Round Addition in .BLIF file with presence of 30 % defective cells after succesful reconfiguration

**Fig. 13:**
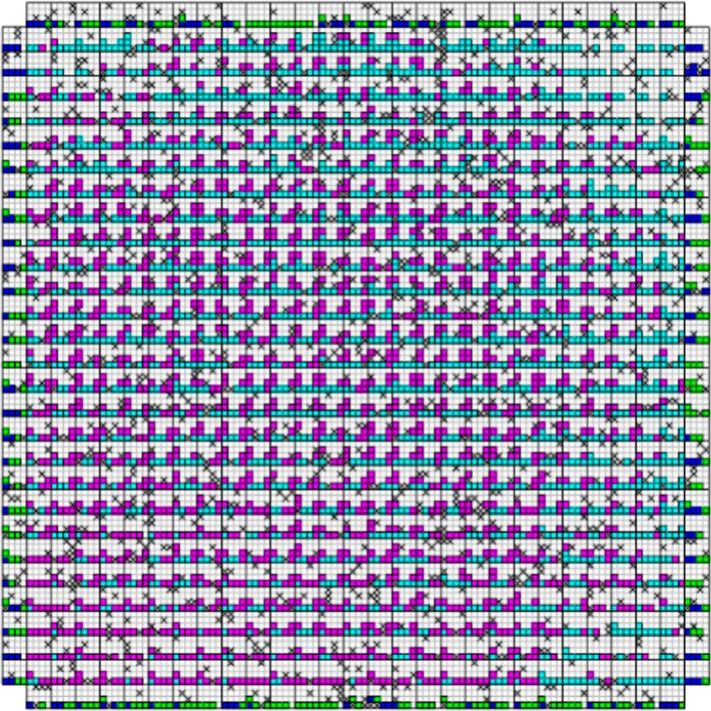
Placement of Round Operation with the peresence of 10 % defective cells on (27 + 2) × (27 + 2) tile array

**Fig. 14:**
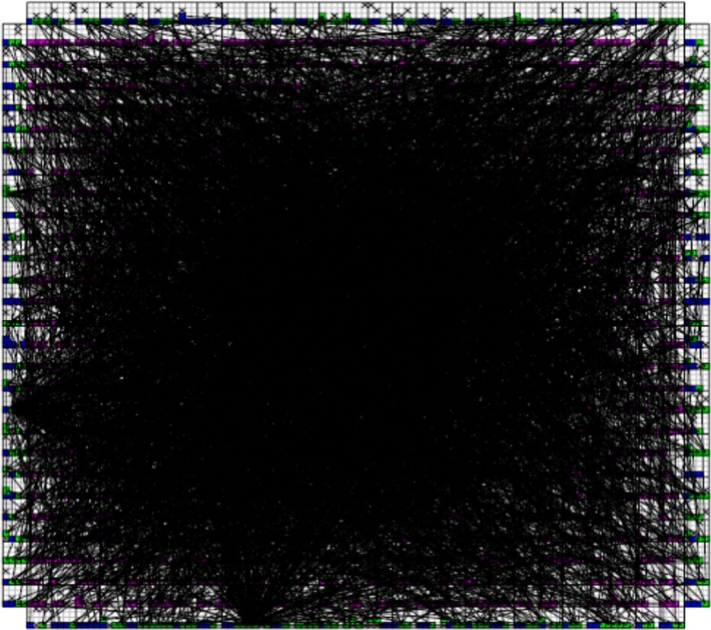
Initial placement of Round Operation with the peresence of 10 % defefective cells on (27 + 2) × (27 + 2) tile array

**Fig. 15:**
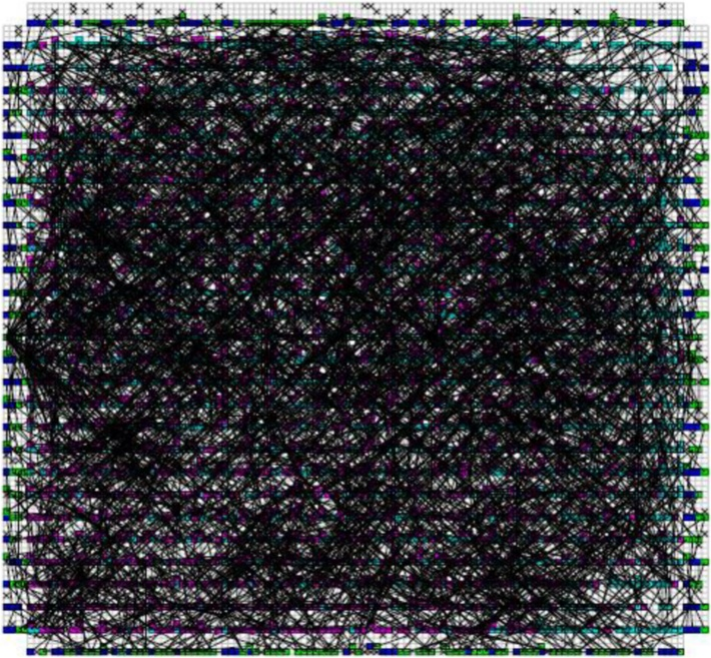
Final routing and palcement of Round Operation with the presence of 10 % defective cells after succesful reconfiguration. The top layer is covered with a high density mesh of nanowires

**Fig. 16:**
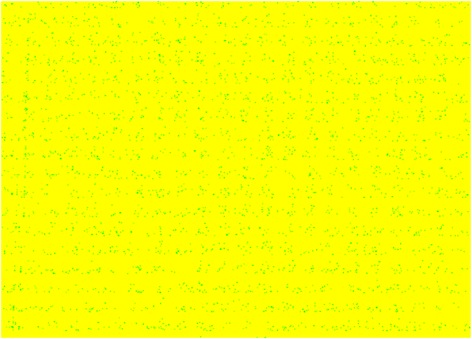
A global view of Round Operation mapped on a hybrid CMOS-Nano fabric. Active nanoelements are identified with green dots. Bad and unused nanoelements are not shown

**Fig. 17:**
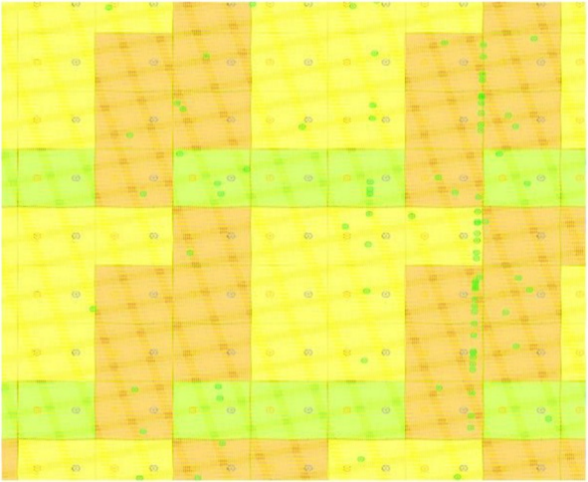
A zoomed view of Round Function mapped on a hybrid CMOS-Nano fabric. Active nanoelements are identified with green dots. Bad or unused nanoelements are not shown

**Fig. 18:**
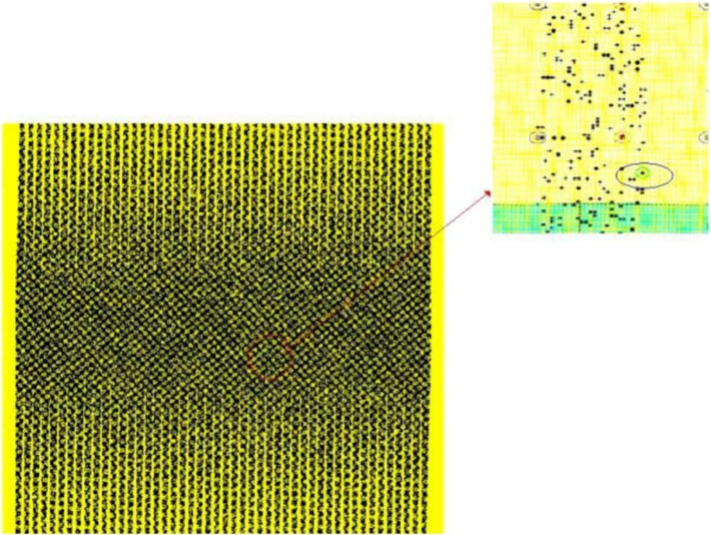
A global view of Round Word Computation mapped on a hybrid CMOS-Nano fabric with 30 % defective cells. Bad nanoelements are shown in black, good used green. 30 % of nanoelements are faulty

With *V*_*DD*_ = 0.3 V and given a 15 nm-wide metallic nanowire interconnects with 3 nm thick switching layer separating two nanowire layers, and an insulator between and around all nanowires with a dielectric constant of 3.9 (that of SiO_2_), the wire capacitance per unit length to be close to 0.2 fF/μm, capacitance *C*_*wire*_ of the full nanowire fragment will be about 3 fF. It is known that *R*_*ON*_ = 400 kΩ and the “ON” resistance of a crosspoint nanodevice is $\frac{R_{ON}}{D}=5\ k\Omega,\ D=80$ is the number of parallel nanodevices connected in series with the ohmic resistance *R*_*wire*_, driven by voltage *V*_*DD*_ and *I* is the maximum gate fanin (http://www.eecg.utoronto.ca/vpr/). The wire resistivity is almost at 8.88 μΩcm and the substrate and coupling capacitances are about 2 pF/cm and 1 pF/cm, respectively. The estimated resistance between the center and the end of a nanowire fragment, of the length $\frac{\beta {\left({F}_{CMOS}\right)}^2}{F_{nano}}=7.2\ \upmu m$ is estimated less than 2.5 kΩ. Assuming the area of the minimum-width transistor to be 25(*F*_*CMOS*_)^2^ (http://www.eecg.utoronto.ca/vpr/), where *F*_*CMOS*_ is the half pitch of the CMOS subsystem, the results for CMOS implementation is shown in the Table 1. Table 2 summarizes the performance estimation for the same circuits on hybrid CMOS-Nano FPGA fabric without defect. Table 3 shows the same with 10 % and 30 % defect rates. As it is seen, the hybrid-CMOS implementation is almost two orders of magnitude denser than its CMOS counterpart. Figure 9 shows the implementation of one columns of *Final Round Addition* on a (9 + 2) × (9 + 2) CMOS FPGA block in which the critical path is represented by green color. Figure 10 shows the initial placement for that circuit in .BLIF format mapped on the (9 + 2) × (9 + 2) tile array with 30 % defects. (Here the additional layer of tiles at the array periphery is used exclusively for I/O functions). Figure 11 show initial placement and routing of the same circuit on the same platform. Figure 12 shows the same circuit after final placement and routing. Figure 13 shows the placement of *Round Operation* with 10 % defective cells on a (27 + 2) × (27 + 2) CMOS-Nano FPGA. Figure 14 shows the initial placement of *Round Operation* with 10 % defective cells. Figure 15 shows final routing and placement of the same circuit after final successful reconfiguration. Figure 16 shows the same implementation in which active nanoelements are shown with green dots. Figure 17 shows a zoomed view of *Round Operation* mapped on a hybrid CMOS-Nano fabric. Active nanoelements are identified with green dots. Bad or unused nanoelements are not shown. Figure 18 shows a global view of *Round Word Computation* mapped on a hybrid CMOS-Nano fabric with 30 % defect rate. Bad nanoelements are shown in black while good used are shown in green.

**Table 1:** Performance results for SHA-512 building blocks mapped on CMOS FPGA

Circuit	CMOS FPGA				
	F_CMOS_ = 45 nm				
	Depth	LUT	Linear size	Area (*μm* ^2^)	Delay (ns)
Round function	1	1218	8 × 8	124538	6.7
Round operation	67	2769	27 × 27	304722	39.7
Final round addition	19	286	9 × 9	36320	22
Round word computation	7	1440	20 × 20	151369	3.7

**Table 2:** Performance Results For SHA-512 Building blocks mapped on two-cell hybrid CMOS-NANO FPGA fabric

Circuit	Hybrid CMOS/NanoDevice FPGA				
	F_CMOS_ = 45 nm, F_nano_ = 4.5 nm, Max fan in = 7				
	Depth	Tile size	No. of nano devices	Area (μm^2^)	Delay
Round function	23	12 × 12	2309	299	1.76
Round operation	111	25 × 25	5786	1296	14.2
Final round addition	67	7 × 7	602	168	10.6
Round word computation	14	23 × 23	2146	1096	1.6

**Table 3:** Performance results for SHA-512 building blocks mapped on two-cell hybrid CMOS-NANO FPGA with two different defect rates

Circuit	No defect		10 % defective cells		30 % defective cells	
	Area	Delay (ns)	Area (*μm* ^2^)	Delay (ns)	Area (*μm* ^2^)	Delay (ns)
Round function	299	1.76	351	1.76	407	1.79
Round operation	1296	14.2	1512	15.68	2540	17.24
Final round addition	102	10.56	102	10.56	168	10.6
Round word computation	1096	1.6	1096	1.8	1195	1.92

**Fig. 19:**
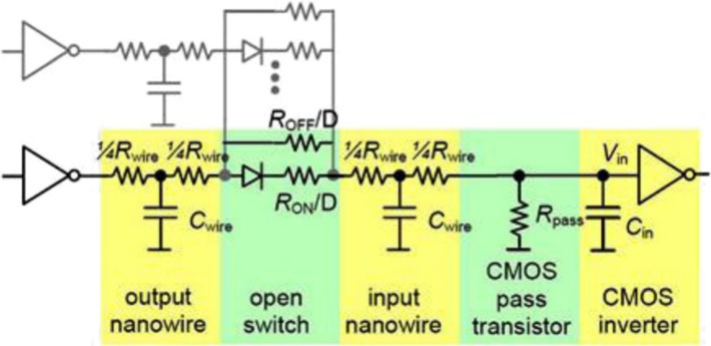
Equivalent circuit of hybrid CMOS-Nano logic stage [30]

Static power consumption of two cell hybrid CMOS-Nano circuits can be estimated as the sum of static power consumption *P*_*ON*_ due to currents *I*_*ON*_^*,*^, leakage power consumption *P*_*leak*_ due to current leakage through nanodevices in their “OFF” state [24, 25]. Figure 19 shows the equivalent circuit for hybrid CMOS-Nano logic stage.

2 $$ {P}_{ON} \simeq \frac{V_{DD}^2}{2\ {R}_{ser}},\ {P}_{leak} \simeq \frac{M{V}_{DD}^2}{2\frac{R_{OFF}}{D}} $$

where *R*_*ser*_ is the series resistance equivalent of *R*_*ON*_ */D* and *R*_*wire*_, *D* is the total number of nanoscale switches in one nanowire crosspoint, *M* is the number of closed crosspoint switches. With *F*_*CMOS*_ = 45nm*, F*_*nano*_ = 4.5nm, *R*_*wire*_ = 14Ω, *R*_*OFF*_ = 4 *G*Ω, and *D* = 80 [24], the leakage and static power consumption of each module can be estimated. It is clear that P_*leak*_ can be neglected. Dynamic power consumption *P*_*d*_ is mainly due to recharging of nanowire capacitances and depends on the number of nanowires allocated by the synthesis tool to implement a circuit onto the target platform and can be calculated using Eq. (3).

3 $$ {P}_d = \frac{1}{2}\ \alpha NC{V}_{dd}^2f $$

Where *α* is the average ‘switching activity’ of the circuit, *N* is the number of nanowires participating in the implementation of logic function, *C* is the capacitance of single nanowire, *V*_*dd*_ is the voltage supply of CMOS transistors, and *f* is the maximum clock speed determined by timing analysis of critical path. We chose an activity of 0.2, twice of the value predicted by Davis [26] to estimate the power consumption pessimistically not optimistically. Hence, the power consumption of each module (without defect) can be estimated as Table 4.

**Table 4:** Power consumption of SHA-512 building blocks mapped on two-cell hybrid CMOS-NANO FPGA

Circuit	Hybrid CMOS/NanoDevice FPGA	
	F_CMOS_ = 45 nm, F_nano_ = 4.5 nm, Max fan in = 7	
	Static power consumption (mW)	Dyanamic power consumption (mW)
Round function	0.7	16
Round operation	6.5	19
Final round addition	0.7	2.7
Round word computation	0.9	16

We have not computed the total power consumption of the whole algorithm; however, evidences show that the power consumption of the proposed design is close and comparable to the power consumption of the actual implementation of the algorithm on FPGA [27].

## Conclusions

The invention of the transistor is one of the most important inventions of the 20^th^ century. Since its inception, the transistor size has been reduced so that now modern devices are orders of magnitude smaller than their earliest counterparts. Unfortunately, the scaling down will eventually end. Increasing power, capital costs, and ultimately theoretical size limitations, are poised to halt the process of continually shrinking the transistor. The results presented in this paper clearly demonstrate that nanoelectronic-based digital circuits may continue the performance scaling of microelectronics well beyond the limits of the currently dominating CMOS technology. However, whether nanoelectronics will be a replacement for conventional ICs, or as a complimentary technology, is yet to be investigated. We believe that this situation may justify large-scale research and development efforts in this area. In this paper, we discussed the contribution that nanotechnology may offer to the evolution of cryptographic hardware and embedded systems and demonstrated how nanoelectronics can be used for constructing security primitives. There are still some problems but the prospect of cheaply integrating 10^12^ devices per chip is a powerful incentive to overcome the existing challenges. In order for this prediction to become true, several challenges still have to be overcome. Without a doubt, the most important of them is the development of a highly reproducible technology for VLSI fabrication of crosspoint resistive switches. Finally, the preliminary research indicates that while existing parts of the CAD tools will be useful for nano-electronics, there will need to be some additions and changes made. Improved device models and 3-D CAD and design tools will certainly accelerate research in this area.
